# CD20-negative primary middle ear diffuse large B-cell lymphoma coexpressing MYC and BCL-2 secondary to acute lymphoblastic leukemia: A case report

**DOI:** 10.1097/MD.0000000000015204

**Published:** 2019-04-12

**Authors:** Chao Ding, Ying Huang, Mingxia Shi, Bo Nie, Yuntao Li, Kun Wu, Jinrong Yang, Yun Zeng

**Affiliations:** Department of Hematology, The First Affiliated Hospital, Kunming Medical University, Kunming, Yunnan Province, China.

**Keywords:** CD20 negative, diffuse large B-cell lymphoma, double expression, poor prognosis, second neoplasms

## Abstract

**Rationale:**

Second diffuse large B-cell lymphoma (DLBCL) after treatment of acute lymphoblastic leukemia (ALL) is uncommon. To our knowledge, primary middle ear DLBCL which presents CD20-negative and coexpression of MYC and BCL-2 has not been reported yet.

**Patient concerns:**

A 20-year-old Chinese man complained fever and weakness for 2 months. Subsequently bone marrow morphology and flow cytometry immunophenotype suggested ALL. Administrated with 9 cycles of multiagent combined chemotherapy, he felt right ear progressive hearing loss, otalgia, aural fullness. Otoendoscopic examination revealed a pitchy mass obstructing the right external auditory canal. Then the mass resection was performed for biopsy and immunohistochemistry examination.

**Diagnosis:**

The mass was diagnosed as DLBCL which was negative for CD20 and double expression of MYC and BCL-2.

**Interventions:**

Chemotherapy.

**Outcomes:**

The patient eventually gave up and died of severe infection.

**Lessons:**

Although intensive chemotherapy has markedly improved the survival of ALL, more and more secondary cancers have been reported. In addition, primary middle ear lymphoma is much rare; hence, it is easy to be misdiagnosed. Furthermore, DLBCL with negative CD20 and double expression of MYC and BCL-2 is aggressive, which is characterized by chemotherapy resistance and inferior survival rates. We discuss this case aiming at raising awareness of tumors secondary to ALL and exploring the appropriate treatment options for the rare DLBCL.

## 1 Introduction

Acute lymphoblastic leukemia (ALL) is a malignant tumor originating from lymphoblastic precursor cells. There are 2 onset peaks for ALL, 1 is in childhood (5.3/100,000) and the other is over 80 years old (2.3/100,000).^[1]^ The 5-year overall survival (OS) in children is 90%, while 30% to 40% in adults with conventional cytotoxic chemotherapy.^[2]^ Though pretty good prognosis in children ALL, the secondary neoplasms should not be ignored. According to a multicenter retrospective analysis, the probability of non-Hodgkin lymphoma (NHL) secondary to childhood and adolescent ALL was approximately 0.1%, and 5-year survival rates were 68.5 ± 6.4%.^[3]^ Moreover, CD20-negative NHLs account for a rate of 1% to 2% of all B-cell NHLs^[4]^ with highly aggressive pathologies, high levels of chemotherapy resistance and low survival rates which pose significant diagnostic and treatment challenges.^[5]^

Herein we report a case of secondary CD20-negative diffuse large B-cell lymphoma (DLBCL) coexpressing MYC and BCL-2, which originated in middle ear. To the best of our knowledge, this is the 1st case reported in the literature. Due to the rarity, aggressiveness and poor prognosis, the lymphomas need more attention.

## 2 Case report

**Figure 1:**
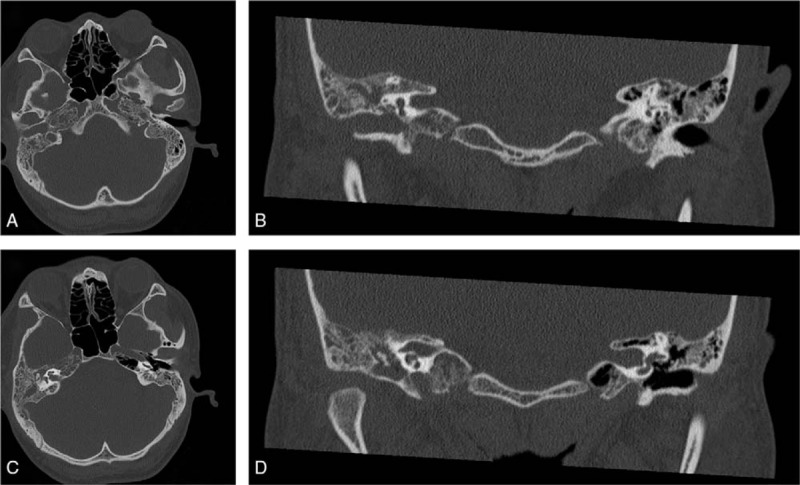
Axial (A) and coronal (B) computed tomography (CT) images showed a soft-tissue density occupying the right external auditory canal. Axial (C) and coronal (D) CT images showed occupation of the right mastoid antrum, tympanic cavity, embedment of the right ossicle, and no significant temporal bone destruction.

**Figure 2:**
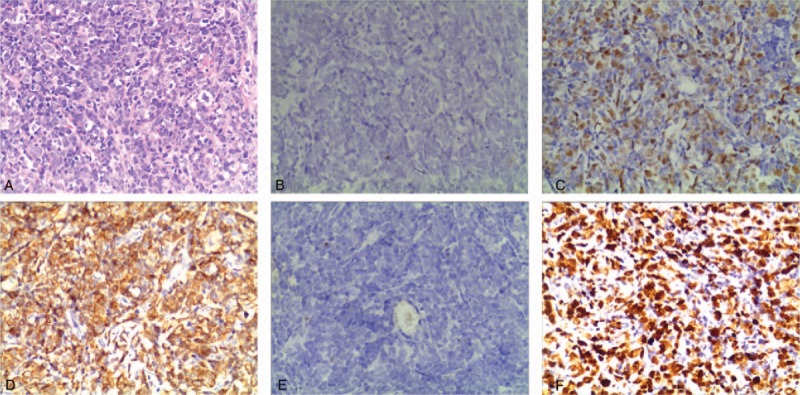
Pathologic images of the diffuse large B-cell lymphoma. Hematoxylin and eosin staining is shown in A (×20). The cell immunohistochemical stainings were negative for CD20 (B, ×20), BCL-6 (E, ×20), positive for MYC (C, ×20), BCL2-2 (D, ×20), and high KI-67 index (F).

A 20-year-old Chinese man complained fever, cough, and weakness for 2 months. In May 2016, he was referred to our hospital. The patient had no family history of malignancy. Blood routine examination indicated severe anemia (hemoglobin 42 g/L), visible immature cells (12%). There was 72% blasts in bone marrow aspirate and flow cytometric analysis revealed a population of abnormal cells (86.53%) with immunophenotype of CD19^+^, cCD79a^+^, CD34^+^, HLA-DR^+^, TDT^+^, CD10^+^ (partially), dimCD22^+^, dimCD33^+^, CD20^−^, cCD3^−^, CD7^−^, which suggested ALL (common B-ALL). As the chromosome was normal and no BCR/ABL fusion gene was found, he was diagnosed with Philadelphia chromosome-negative ALL. Then he was treated with a cycle of VDCP (vincristine, doxorubicin, cyclophosphamide, prednisolone)-like induction chemotherapy. At the end of 1st cycle, the bone marrow minimal residual disease (MRD) was <0.01%, which indicated molecular complete remission (CR). Then he was administrated 2 courses of HD-MTX (high-dose methotrexate), 1 course HD-MTX plus l-asparaginase, 3 courses of CAM (cyclophosphamide, cytarabine, 6-mercaptopurine), 1 course of MA (mitoxantrone, cytarabine) as consolidation chemotherapy, and 6 intensive intrathecal injections of methotrexate, dexamethasone, and cytarabine to prevent central nervous system (CNS) infiltration. During this period, bone marrow morphology or MRD all suggested molecular CR. In December 2017, he felt right ear progressive hearing loss, otalgia, aural fullness. Hospitalized in Department of Otolaryngology in January 2018, oto-endoscopic examination revealed a pitchy mass occluding the right external auditory canal (EAC) and tympanic membrane was not visible. Pure tone audiometry showed a right conductive hearing loss. The temporal bone computed tomography (CT) scan showed a soft-tissue density occupying the right EAC, middle ear, and mastoid antrum (Fig. 1). Then a mass excisional biopsy was performed, the histologic examination indicated a small round cell tumor. The immunohistochemistry (IHC) analysis was positive for MYC (45%), BCL-2 (70%), CD10, CD79a, PAX-5, and negative for MUM-1, BCL-6, CD3, CD20 (repeated 3 times), and CD5 (Fig. 2). The immune-proliferative activity (Ki-67 index) was about 80%. The patient was diagnosed with CD20-negative DLBCL coexpressing MYC/BCL-2. According to the Hans classifier,^[6]^ it was classified as germinal center B (GCB) type. Given the lack of CD20 expression, specimens were sent to the West China Hospital, Sichuan University for pathology consultation. They obtained similar IHC staining with a higher Ki-67 index (90%), MYC (70%), and BCL-2 (80%). Moreover, MYC, BCL-2, and BCL-6 translocation was negative in fluorescence in situ hybridization examination. He was hospitalized again in the Department of Hematology 1 month later in February 2018. Bone marrow morphology was CR for ALL. Laboratory examination showed elevated erythrocyte sedimentation rate 71 mm/h (normal: 0–15 mm/h). The lactate dehydrogenase, β2-microglobulin, human immunodeficiency virus (HIV) showed no abnormality. It was a pity that the patient was not examined by positron emission tomography–CT and we were unable to determine the disease stage accurately. The patient was administrated a course of EPOCH (etoposide, cyclophosphamide, doxorubicin, vincristine, prednisone)-like chemotherapy regimen. He eventually gave up and died of severe infection in June 2018.

## 3 Discussion

With improved survival rate, it is necessary to evaluate the treatment efficacy on secondary cancers in ALL survivors. Multicenter retrospective studies showed the incidence of second malignant neoplasms among childhood and adult ALL ranges 0.5% to 1.6%. The second cancers includes acute myeloid leukemia (AML), myelodysplasia (MDS), NHL, and other solid tumors such as brain tumor, thyroid cancer, basal cell carcinoma, lung cancer, etc.^[3,7–10]^ Hematologic malignancies develop relatively early, while solid cancers have a longer latency period.^[7]^ Causal factors to be considered are genetic predisposition, the type of previous chemotherapy and/or radiotherapy and their cumulative dose. Alkylating agents and more recently DNA-topoisomerase II inhibitors (i.e., etoposide, mitoxantrone, idarubicin) have been linked to the development of secondary AML and MDS.^[11,12]^ A major feature of topoisomerase II inhibitors is the generation of DNA double-strand breaks, the repair of which can readily form substrates for inappropriate exchange which may be a risk factor for 2nd NHL. Another retrospective study in adults ALL failed to demonstrate a clear relationship between the dose of cyclophosphamide or anthracyclines and the occurrence of secondary neoplasms, because multiagent chemotherapy, as part of multimodality therapy for malignant disease, has increased the difficulty to assess which agents might play a causative role in the development of secondary neoplasms.^[9]^ Until now there is no consistent agreement on whether chemotherapy agent would be a predisposition factor for secondary NHL. Even though several studies have documented a clear relationship between prior irradiation therapy and the occurrence of 2nd CNS tumors,^[13]^ sarcomas of bone,^[14]^ and thyroid cancer,^[15]^ there was still no evidence of increased risk of secondary NHL even among the most studied group of atomic bomb survivors in Japan.^[16]^ The association of radiotherapy with secondary NHL is difficult to confirm, in contrast to the association with solid tumors.

As we all know, cancer is at its root a genetic disease resulting from the accumulation of mutations that deregulate cellular differentiation, proliferation, and/or survival. The man we report seemed to predispose to hematopoietic malignancies, so there was a question whether he developed a cancer genetic susceptibility syndrome. As a matter of fact, leukemia and lymphomas are seen in association with a number of cancer genetic susceptibility syndromes, and it is estimated that about 2% to 4% of patients with hematopoietic malignancies develop the disease as a result of an underlying predisposition.^[17,18]^ Cancer genetic susceptibility syndromes are strongly related to family cancer history, presenting features of the tumor and histology, physical examination, or cognitive/developmental manifestation and are associated with risk for hematopoietic cancers.^[19]^ Thereinto, constitutional mismatch repair deficiency (CMMRD) syndrome is related with DNA mismatch repair genes PMS2, MSH6,MSH2, or MLH1. Though poor prognosis, no established and efficient surveillance options are available in CMMRD.^[20]^ We did not consider this rare disease with the patient, as he had no family history of malignancy and had normal cognitive/developmental manifestation.

Extranodal involvement may be present in as many as 30% patients with NHL. The incidence of gastrointestinal tract is the 1st followed by head and neck region.^[21]^ Primary middle ear lymphoma is very rare, which often present otalgia, moderate to severe conductive hearing loss, low grade fever and facial paralysis.^[22]^ It is a question where the lymphoma origin from, as the normal middle ear cavity does not contain lymphoid tissue. A report suggested that a layer of lymphoid tissue located deep to the epithelium of the mucosa lining the mastoid antrum, tympanic cavity, and tympanic orifice of the eustachian tube acts as the site of origin of the primary lymphoma.^[23]^ CT/magnetic resonance imaging is essential for highly suspected ear lymphoma. Of course, a definitive diagnosis should always be based on pathologic examinations.

The DLBCL is characterized by medium- to large-sized neoplastic cells that express a wide range of B-cell markers including CD19, CD20, CD22, and CD79a.^[24]^ Since CD20-negative DLBCL was reported,^[25]^ several mechanisms for the CD20 phenotypic change after rituximab administration have been proposed, including selection of a CD20-negative clone as a consequence of rituximab exposure or downregulation of the CD20 antigen by genetic and epigenetic changes.^[26]^ As an extremely rare disease, CD20-negative DLBCLs include primary effusion lymphoma (PEL), plasmablastic lymphoma (PBL), anaplastic lymphoma kinase (ALK)-positive DLBCL, and large B-cell lymphoma arising from human herpesvirus 8 (HHV-8)-associated multicentric Castleman disease (MCD).^[27]^ They all commonly express markers of plasmacytic differentiation such as CD38, CD138, or MUM-1. The malignancy is commonly related to virus infection such as HIV, HHV-8, and/or Epstein–Barr virus except ALK^+^ DLBCLs, which are characterized by the chromosomal translocation of the ALK gene to other partner genes (i.e., NPM, CLTC).^[28]^ According to an analysis of the National Cancer Data Base on CD20-negative DLBCL, with a median follow-up time of 2.6 years, median OS was 5 months for PEL, 15 months for PBL, 13 months for ALK^+^ DLBCL, and it was not reached for HHV-8+DLBCL.^[29]^ Another retrospective study reported the median survival time was <2 months, and the 1-year OS rate was only 24% for HHV-8+DLBCL.^[5]^

Rituximab, a chimeric CD20 monoclonal antibody, was later developed and approved for treatment of human B-cell malignancies. The addition of rituximab to cyclophosphamide, doxorubicin, vincristine, and prednisone has dramatically improved the survival of patients with DLBCL.^[30]^ Given the absence of CD20 expression, CD20-negative DLBCLs cannot benefit from rituximab administration and pose a therapeutic dilemma. Until now there is still no standard of care for CD20-negative DLBCL. CODOX-M/IVAC (cyclophosphamide, vincristine, doxorubicin, methotrexate alternating with ifosfamide, etoposide, cytarabine), dose-adjusted EPOCH and HyperCVAD (cyclophosphamide, vincristine, doxorubicin, and dexamethasone alternating with HD-MTX and cytarabine) are the suggested therapies.^[4]^ Novel agents such as proteasome inhibitor bortezomib for PBL,^[31]^ brentuximab for PEL,^[32]^ anti-interleukin-6 monoclonal antibody siltuximab for MCD^[33]^ and crizotinib for ALK^+^ DLBCL^[34]^ might be promising.

Double-expression lymphoma (DEL) exhibits coexpression of BCL-2/MYC on IHC.^[35]^ Approximately 19% to 34% of DLBCL expressed both MYC and BCL-2 protein.^[36,37]^ In various retrospective studies, DEL more frequently had non-GCB type.^[35,38,39]^ Compared with patients with DLBCL that did not have overexpression of MYC and BCL2, patients with DEL had worse performance status, more advanced stage, multiple extranodal sites, higher international prognostic index, and higher proliferation index.^[40]^ In addition, patients with DLBCL with coexpression of BCL-2 and MYC but BCL-6 negative expression had a more worse prognosis compared with the patients with coexpression of BCL-2, BCL-6, and MYC.^[41]^ Though no standard treatment for DEL, rituximab plus dose-adjusted EPOCH regimen has shown promising preliminary results.^[42]^ One of new promising targets for patients with DLBCL with BCL-2 and MYC coexpression is heat shock protein H1/105, knockdown of which in a lymphoma mouse model downregulated MYC, BCL-2, and BCL-6 expression, inhibited lymphoma cells proliferation and decreased neoangiogenesis.^[43]^

To our knowledge, there are no reports on CD20 negative lymphoma with BCL-2 and MYC double expression. As it is poor prognosis for both CD20 negative DLBCL and DLBCL with dual expression of BCL-2 and MYC, CD20-negative DLBCL with BCL-2 and MYC double expression is expected to have a worse prognosis. Dose-adjust EPOCH regimen may be promising for the new type DLBCL.

## 4 Conclusion

Second NHL as a late side effect of ALL is almost rare. It urges us to pay more attention to the treatment toxicity and to extend the follow-up time. Given no standard regimen and poor prognosis for CD20-negative DLBCL and DEL, we present this case with detailed clinical and pathologic features to increase awareness concerning the rare malignancy among pathologist and clinicians to search more effective treatment strategy to improve the survival rate.

## Acknowledgment

Most sincere thanks are given to pathologists and imaging specialist of the First Affiliated Hospital, Kunming Medical University for their kindness of providing all the pathologic and CT images.

## Author contributions

**Conceptualization:** Yun Zeng.

**Data curation:** Yuntao Li.

**Formal analysis:** Chao Ding, Mingxia Shi.

**Funding acquisition:** Yun Zeng.

**Investigation:** Chao Ding.

**Methodology:** Chao Ding, Bo Nie.

**Project administration:** Chao Ding, Mingxia Shi, Bo Nie.

**Resources:** Ying Huang.

**Software:** Kun Wu.

**Supervision:** Jinrong Yang.

**Visualization:** Mingxia Shi.

**Writing – original draft:** Chao Ding.

**Writing – review & editing:** Yun Zeng.
